# Antibacterial activity of *Nocardia* spp. and *Streptomyces* sp. on multidrug-resistant pathogens causing neonatal sepsis

**DOI:** 10.1590/S1678-9946202466042

**Published:** 2024-07-29

**Authors:** Janette Berenice González-Nava, Gauddy Lizeth Manzanares-Leal, Luis Ángel Zapi-Colín, Sonia Dávila-Ramos, Horacio Sandoval-Trujillo, Ninfa Ramírez-Durán

**Affiliations:** 1 Universidad Autónoma del Estado de México, Facultad de Medicina, Laboratorio de Investigación en Microbiología Médica y Ambiental, Toluca, Mexico; 2 Universidad Autónoma del Estado de México, Facultad de Medicina, Laboratorio de Neuroquímica, Toluca, Mexico; 3 Universidad Autónoma del Estado de Morelos, Instituto de Investigaciones en Ciencias Básicas y Aplicadas, Centro de Investigación en Dinámica Celular, Morelos, Mexico; 4 Universidad Autónoma Metropolitana, Departamento de Sistemas Biológicos, Laboratorio de Producción de Biológicos, Ciudad de México, Mexico

**Keywords:** Neonatal sepsis, PKS, NRPS, Actinobacteria, Antimicrobial drug resistance

## Abstract

Neonatal sepsis leads to severe morbidity and occasionally death among neonates within the first week following birth, particularly in low- and middle-income countries. Empirical therapy includes antibiotics recommended by WHO. However, these have been ineffective against antimicrobial multidrug-resistant bacterial strains such as *Klebsiella* spp, *Escherichia coli*, and *Staphylococcus aureus* species. To counter this problem, new molecules and alternative sources of compounds with antibacterial activity are sought as options. Actinobacteria, particularly pathogenic strains, have revealed a biotechnological potential still underexplored. This study aimed to determine the presence of biosynthetic gene clusters and the antimicrobial activity of actinobacterial strains isolated from clinical cases against multidrug-resistant bacteria implicated in neonatal sepsis. In total, 15 strains isolated from clinical cases of actinomycetoma were used. PCR screening for the PKS-I, PKS-II, NRPS-I, and NRPS-II biosynthetic systems determined their secondary metabolite-producing potential. The strains were subsequently assayed for antimicrobial activity by the perpendicular cross streak method against *Escherichia fergusonii* Sec 23, *Klebsiella pneumoniae* subsp. *pneumoniae* H1064, *Klebsiella variicola* H776, *Klebsiella oxytoca* H793, and *Klebsiella pneumoniae* subsp. *ozaenae* H7595, previously classified as multidrug-resistant. Finally, the strains were identified by 16S rRNA gene sequence analysis. It was found that 100% of the actinobacteria had biosynthetic systems. The most frequent biosynthetic system was NRPS-I (100%), and the most frequent combination was NRPS-I and PKS-II (27%). All 15 strains showed antimicrobial activity. The strain with the highest antimicrobial activity was *Streptomyces albus* 94.1572, as it inhibited the growth of the five multidrug-resistant bacteria evaluated.

## INTRODUCTION

Neonatal sepsis is an infection produced by some bacteria, fungi, or viruses present in normally sterile body fluids. This condition causes hemodynamic changes whose clinical manifestations range from subclinical to severe systemic infections. The pathology is classified into early-onset neonatal sepsis, when the clinical manifestations occur in the first three days of life, in which case it is assumed that perinatal infections occurred; into late-onset neonatal sepsis, which manifests from the fourth day to 30 days of age; or into very late-onset neonatal sepsis, which is diagnosed after 30 days of age until patient discharged. In the latter two cases, the infection is usually acquired in the hospital or the community^1,2^.

Neonatal sepsis is the third leading cause of neonatal death, after preterm birth and birth complications. Its incidence varies depending on the population, from 1 to 5 cases per 1,000 live births^1^. It is estimated to cause 2.6 million newborn deaths per year worldwide. Most of these deaths occur in low- and middle-income countries, with Africa, Asia, and Latin America presenting the highest prevalence of this pathology^1,3,4^. The most frequent causative agents of neonatal sepsis are some species of *Klebsiella* spp., *Pseudomonas* spp., *Enterobacter* spp., *Candida* spp., *Staphylococcus aureus, Staphylococcus epidermis*, and *Escherichia coli* ^1,5,6^.

As in other infections, antimicrobial resistance is alarming and of concern in treating neonatal sepsis^5,6-8^. Species of *Klebsiella* sp., *Escherichia* sp., and *Pseudomonas* sp. have developed various genetic mechanisms to resist antibiotics^9^. Currently, microorganisms associated with neonatal sepsis have been detected with multidrug resistance (MDR) , as well as extensively drug-resistant (XDR) and pandrug-resistant (PDR) patterns^5,10^. To counteract this problem, the Global Antibiotic Research and Development Partnership (GARDP) promotes the search for and development of new antibiotics intending to have therapeutic alternatives for infections caused by antimicrobial-resistant microorganisms^6,11^.

Actinobacteria stands out as one of the alternatives, since these microorganisms produce bioactive compounds such as antibiotics, antifungals, or anticancer agents^6^. Some commonly used antibiotics from actinobacteria are erythromycin, kanamycin, streptomycin, tetracycline, and vancomycin. The genus *Streptomyces* is the major producer of antibiotics in the Actinomycetaceae family; however, other actinobacteria with production potential of bioactive compound have been reported as novel antibiotics^12,13^.

Actinobacteria production capacity of bioactive compound is determined by biosynthetic gene clusters (BGCs) within their genome. BGCs are a set of genes that encode catalytic enzymes capable of synthesizing bioactive molecules with diverse chemical structures, which can be used as antibiotics, anticancer agents, immunosuppressants, cholesterol reducers, food additives, phytosanitary agents, and others. BGCs can be classified into polyketides, non-ribosomal peptides, saccharides, alkaloids, and terpenoids. The BGCs called polyketide synthase (PKS) and non-ribosomal peptide synthases (NRPS) can be classified into three types: PKS-I, PKS-II, and PKS-III; NRPS-I, NRPS-II, and NRPS-III, respectively^14^.

Several genomic studies have shown that, within the actinobacteria, the number of biosynthetic genes of the genus *Nocardia* is comparable to *Streptomyces* ^12,13^. Likewise, it has been described that actinobacteria can be isolated from insufficiently studied environments such as mangroves, marine sediments, or clinical cases of actinomycetoma^13^. Hence, this study aimed to determine the presence of biosynthetic gene clusters and the antimicrobial activity of actinobacterial strains isolated from clinical cases against multidrug-resistant bacteria involved in neonatal sepsis.

## MATERIALS AND METHODS

### Actinobacteria

An experimental study was performed using 15 actinobacterial strains obtained from the Instituto de Diagnostico y Referencia Epidemiologica (InDRE) (Institute of Epidemiological Diagnosis and Reference) in Mexico City, Mexico. The strains were isolated from clinical cases of actinomycetoma from different body sites such as the foot, thorax, back, face, leg, dorsum, buttock, neck, lung, knee, sternum, and arm. They were previously classified within the Actinomycetaceae family via biochemical tests with xanthine, hypoxanthine, lysozyme resistance, tyrosine, and casein degradation.

### Multidrug-resistant bacteria

For the analysis of antimicrobial activity, five multidrug-resistant bacteria were included: *Escherichia fergusonii* Sec23, isolated from an injury discharge^15^; *Klebsiella oxytoca* H793, isolated from a case of neonatal pneumonia^16^; and *Klebsiella pneumoniae* subsp. *pneumoniae* H1064; *Klebsiella variicola* H776; as well as *Klebsiella pneumoniae* subsp. *ozaenae* H759, isolated from neonates diagnosed with sepsis^15,16^ (Table 1), all cases were treated in an intensive care unit.

**Table 1:** Origin of multidrug-resistant strains included in the study^15^,^16^.

Strain code	Bacterial species	Type of sample	Age	Diagnosis of patient
Sec23	*Escherichia fergusonii*	Injury discharge	14 days	Injury with abscess
H793	*Klebsiella oxytoca*	Tracheal aspirate	21 days	Pneumonia
H1064	*Klebsiella pneumoniae* subsp. *pneumoniae*	Blood	21 days	Sepsis
H776	*Klebsiella variicola*	Blood	9 days	Sepsis
H759	*Klebsiella pneumoniae* subsp. *ozaenae*	Blood	12 days	Sepsis

The criteria for diagnosing neonatal sepsis included the presence of abnormal temperature (hypothermia or fever), tachypnea, feeding difficulty, irritability, abnormal skin coloration, white blood cell count (WBC) reduction to < 5 × 109/L, platelet count (PLT) ≤ 100 × 109/L, erythrocyte sedimentation rate ≥ 15 mm/h, and positive blood cultures for pathogenic microorganisms^16^.

As described in previous studies^15,16^, blood culture tubes were incubated at 35 °C and inspected daily for signs of bacterial growth for seven days. This process was repeated in triplicate. Routine subculture was performed 24h, 48h, and seven days. Subsequently, strains were characterized for their level of antimicrobial resistance by broth microdilution and disk diffusion methods, with posterior genetic identification (Table 2). Multidrug-resistant bacteria were defined as those resistant to one or more drugs from more than three antimicrobial families^17^.

**Table 2:** Resistance profile of the multidrug-resistant bacteria included in the study^15^,^16^.

Family	Antibiotic		Strains			
		*E. fergusonii* Sec23	*K. pneumoniae* H1064	*K. variicola* H776	*K. oxytoc a* H793	*K. pneumoniae subsp. ozaenae H759*
Aminoglycosides	Amikacin	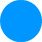	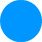	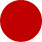	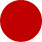	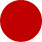
	Gentamicin	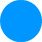	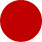	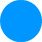	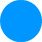	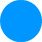
	Netilmicin	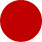	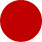	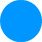	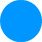	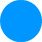
	Tobramycin	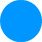	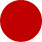	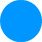	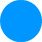	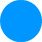
Monobactams	Aztreonam	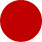	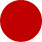	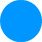	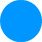	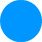
Carbapenemics	Doripenem	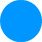	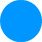	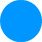	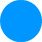	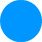
	Ertapenem	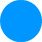	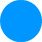	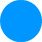	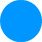	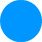
	Imipenem	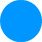	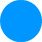	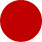	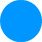	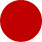
	Meropenem	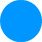	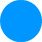	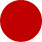	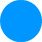	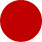
Cephalosporins 1^st^ generation	Cefazolin	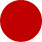	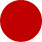	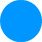	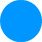	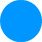
	Cefaclor	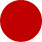	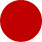	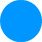	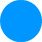	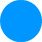
Cephalosporins 2^nd^ generation	Cefotetan	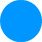	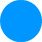	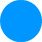	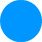	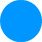
	Cefuroxime	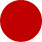	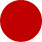	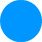	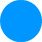	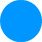
Cephalosporins 3^rd^ generation	Cefoperazone	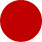	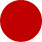	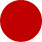	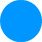	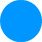
	Cefotaxime	* 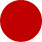	* 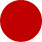	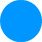	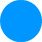	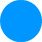
	Cefpodoxime	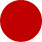	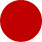	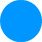	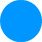	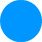
	Ceftazidime	* 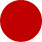	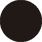	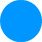	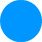	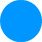
	Ceftibuten	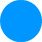	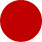	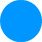	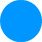	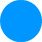
	Ceftriaxone	* 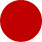	* 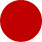	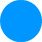	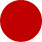	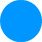
Cephalosporins 4^th^ generation	Cefepime	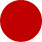	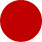	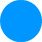	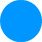	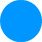
	Ceftaroline	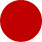	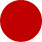	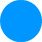	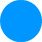	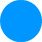
Cephalosporins 3^rd^ / inhibitor	Ceftazidime/avibactam	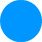	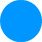	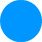	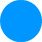	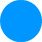
Cephalosporin 5^th^ / inhibitor	Ceftolozane/tazobactam	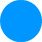	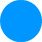	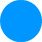	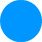	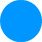
Fenicols	Chloramphenicol	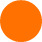	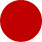	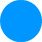	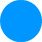	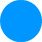
Phosphonates	Fosfomycin	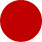	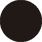	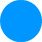	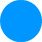	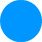
Glycylcyclines	Tigecycline	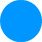	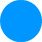	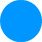	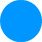	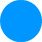
Nitrofurans	Nitrofurantoin	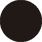	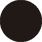	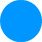	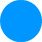	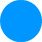
Penicillin	Ampicillin	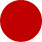	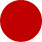	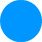	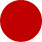	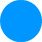
	Piperacillin	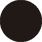	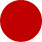	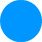	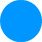	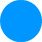
Penicillin/inhibitor	Amoxicillin/clavulanic acid	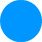	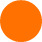	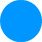	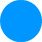	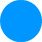
	Ampicillin/sulbactam	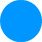	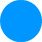	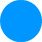	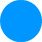	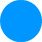
	Piperacillin/tazobactam	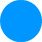	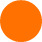	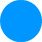	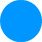	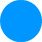
Polymyxins	Colistin	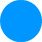	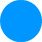	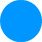	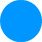	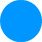
Quinolones 2^nd^ generation	Ciprofloxacin	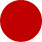	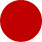	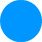	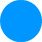	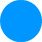
Quinolones 3^rd^ generation	Levofloxacin	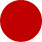	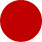	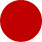	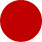	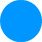
Sulfonamides	Trimethoprim/sulfamethoxazole	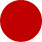	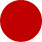	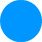	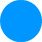	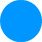
Tetracyclines	Tetracycline	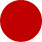	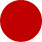	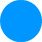	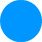	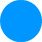

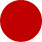
 = resistant; 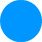 = sensitive; 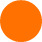 = intermediate; 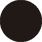 = not determined; *Positive for extended spectrum beta-lactamase (ESBL) production.

### Strains culture

The actinobacteria were inoculated on Sabouraud dextrose agar (Bioxon, cat. 210700) and added with 1% dehydrated potato. They were incubated at 37 °C for 21 days, which corresponds to the average time for the growth of actinobacteria in solid culture media. Multi-resistant strains were inoculated on McConkey agar (BD-BBL, cat. 211662) and incubated at 37 °C for 24h.

### Detection of biosynthetic systems in actinobacteria

The presence of the biosynthetic systems PKS-I, PKS-II, NRPS-I, and NRPS-II was detected by PCR using specific primers. For this purpose, pure strains were inoculated in Sabouraud dextrose liquid medium (Bioxon, cat. 222400) incubated at 37 °C with agitation at 160 rpm for 30 days, which corresponds to the average time for actinobacteria to grow in liquid medium. They were then placed in 1.5 mL tubes with sterile saline solution, and then centrifuged at 1,000 rpm for 5 min to obtain biomass. The supernatant was decanted.

DNA extraction was performed from the biomass obtained following the protocol of the Wizard^®^ genomic DNA purification kit (Promega, cat. A1120), according to the manufacturer’s specifications. DNA integrity was analyzed by electrophoresis on a 1% agarose gel stained with ethidium bromide under the following conditions: 120 V, 300 mA, 50 W for 30 min, using a 1 kb molecular marker (Axygen, cat.m-dna-1kb).

Once DNA was obtained from the strains, the PKS-I and NRPS-I systems were detected by multiplex PCR. The NRPS II and PKS-II systems were amplified by individual PCR. For this purpose, Taq DNA polymerase (Mytaq, bioline cat bio-21105) and specific primers were used, as detailed in Table 3. Agarose gel electrophoresis was performed to observe the amplification product. The conditions are specified in Table 3.

**Table 3:** - PCR conditions for detection of PSK and NRPS biosynthetic systems, 16S rRNA gene and agarose gel electrophoresis.

Biosynthetic system	Primers	Amplicon size (bp)	PCR conditions	Electrophoresis conditions	Article
PKS-I	K1f(5’-TSAAGTCSAACATCGGBCA-3’) M6r (5’-CGCAGGTTSCSGTACCAGTA-3’)	1200–1400	Denaturation at 95 °C for 5 min, and 34 cycles of 95 °C for 30s, 55 °C for 2 min, 72 °C for 4 min, and 72 °C for 10 min	1.5% agarose gel, stained with ethidium bromide. Electrophoresis for 40 min at 120 V and 300µA	Ayuso-Sacido *et al*.^18^
PKS-II	KSα (5’-TSGRCTACRTCAACGGSCACGG-3’) KSβ (5’- TACSAGTCSWTCGCCTGGTTC-3’)	1000	Denaturation at 95 °C for 5 min, and 35 cycles of 95 °C for 30s, 58 °C for 20s, 72 °C for 4 min, and 72 °C for 10 min	1% agarose gel, stained with ethidium bromide. Electrophoresis for 40 min at 120 V and 300µA	Ayuso *et al*.^19^
NRPS-I	A3f (5’-GCSTACSYSATSTACACSTCSGG-3’) A7r (5’-SASGTCVCCSGTSCGGTAS-3’)	700–800	Denaturation at 95 °C for 5 min, and 35 cycles of 95 °C for 30s, 59 °C for 2 min, 72 °C for 4 min, and 72 °C for 10 min	1.5% agarose gel, stained with ethidium bromide. Electrophoresis for 40 min at 120 V and 300µA.	Ayuso-Sacido *et al*.^18^
NRPS-II	F(5’-CCGCCCATGGGTGCTCCGCGTGGCGAGCGGACCCGCGC-3’) R(5’-TGCCCCCTCGCCCTGGCCTCTAGATCC)	300	Denaturation at 95 °C for 5 min, and 40 cycles of 95 °C for 3 s, 63 °C for 45 s, 72 °C for 90s, and 72 °C for 10 min	2% agarose gel, stained with ethidium bromide. Electrophoresis for 30 min at 120 V and 300µA	Du and Shen^20^
16S rRNA gen	8f (5’-AGAGTTTGATCMTGGCTCAG-3’) 1492R(5’-TACGGYTACCTTGTTACGACTT-3’)	1400–1500	Denaturation at 94 °C for 5 min, and 40 cycles of 94 °C for 1 min, 59 °C for 30s, 72 °C for 1 min, and 72 °C for 10 min	1% agarose gel, stained with ethidium bromide. Electrophoresis for 30 min at 120 V and 300µA	Yuan et al.^21^

### Evaluation of antimicrobial activity

Actinobacterial strains with at least one of the PKS and NRPS systems were evaluated for antagonistic activity on the group of multidrug-resistant bacteria (*E. fergusonii* Sec 23, *K. pneumoniae* subsp. *pneumoniae* H1064, *K. variicola* H776, *K. oxytoca* H793, and *K. pneumoniae* subsp. *ozaenae* H7595). For this procedure, the perpendicular cross streak method described by Fritz *et al.* ^22^was used, with modifications in the incubation times due to actinobacteria taking, on average, three weeks to grow *in vitro*.

For the test, a 0.5 suspension on the McFarland scale of each actinobacteria was made, after which each strain was inoculated individually on Sabouraud dextrose agar using a vertical streak diametrically across the Petri dish in the central part. The plates were incubated at 37 °C for 37 days, which is the average time for the actinobacteria to produce secondary metabolites. Subsequently, the multidrug-resistant bacteria were seeded using lines perpendicular to the central line and incubated at 37 °C for 24h. The growth inhibition of the multidrug-resistant strains was noted.

A zone of inhibition of at least 0.1 cm was taken as positive for antimicrobial activity, in agreement with reports comparing the minimum inhibition halo as indicative of actinobacteria activity compared to no halo for negative results^23^.

### Molecular identification of actinobacteria

For molecular identification of actinobacteria, the 16S rRNA gene was sequenced. For this purpose, PCR amplification of the gene was performed. The previously extracted DNA, Taq DNA polymerase (MyTaq, Bioline cat BIO-21105), and the universal primers 8F and 1492R were used. The sequences of the primers used, and the electrophoresis conditions for visualization of the amplified product are detailed in Table 3.

The amplification products were subsequently purified using the PCR Clean-up System kit (Wizard, PROMEGA, A1120), following the manufacturer’s protocol. Subsequently, the amplicons were sent to the sequencing service of MacroGen (Maryland, USA). The sequences obtained were corrected with ChromasPro (version 1.5, Technelysium Pty Ltd, South Brisbane, Queensland, Australia), and then aligned and assembled with BioEdit (version 7.0.5.3). The assembled sequences were compared with sequences of reference strains from the EzBioCLOUD database for final identification.

### Phylogenetic tree

The phylogenetic tree was inferred using the Maximum Likelihood Estimation method and the Tamura-Nei model. The topology with the highest likelihood value was selected, with a branch support calculated with a bootstrap of 1,000 times. The tree includes 30 sequences, 15 belonging to this study and 15 from the NCBI GenBank that were used as reference sequences. Evolutionary analysis was performed with MEGA X^24,25^. The tree was edited in iTOL v 6.6, incorporating the information on the detected systems as well as the bactericidal capacity against the five selected multidrug-resistant strains^26^.

### Biosafety

All procedures were performed following the guidelines of the Laboratory Procedures Manual and the Mexican standard for managing infectious biological waste NOM-087-ECOL-SSA1-2002^27^.

## RESULTS

### Detection of biosynthetic systems

**Figure 1:**
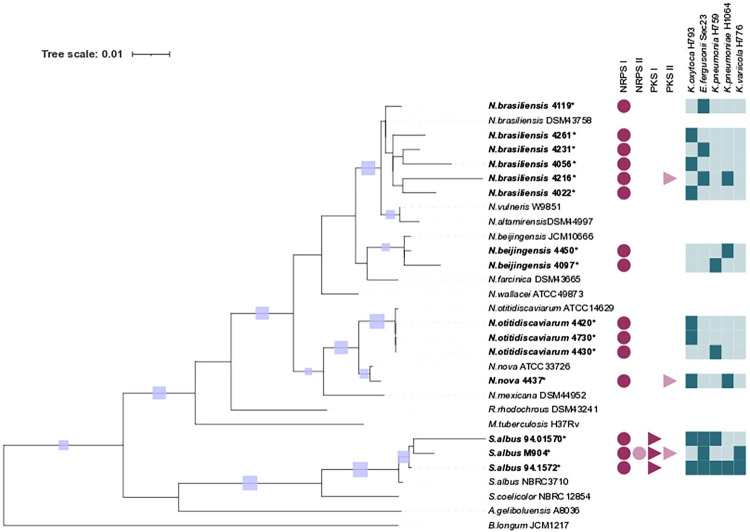
Phylogenetic tree obtained using the ML program with a bootstrap of 1,000 repetitions; support is shown as light blue squares on the branches. The tree shows the evolutionary relationships using the 16S ribosomal gene of 15 strains isolated in this study (Bold type) and 15 reference strains (Regular type). The NRPS-I biosynthetic system is represented in dark pink circles, NRPS-II in light pink circles, PKS-I in dark pink triangles, and PKS-II in light pink triangles. Microbial activity against the multidrug-resistant strains in the upper part is shown in dark blue squares.

At least one biosynthetic system was detected in the 15 evaluated actinobacterial strains. The biosynthetic system with the highest frequency was NRPS-I, which was found in 100% of the strains, and PKS-I and PKS-II which was detected in 20% of the strains. The system with the lowest presence was NRPS-II (6%). Two biosynthetic systems were detected in 27% of the strains, and only one strain (6%) had three biosynthetic systems. As shown in Figure 1, 67% of the strains presented only one biosynthetic system (NRPS-I).

### Detection of antimicrobial activity

**Figure 2:**
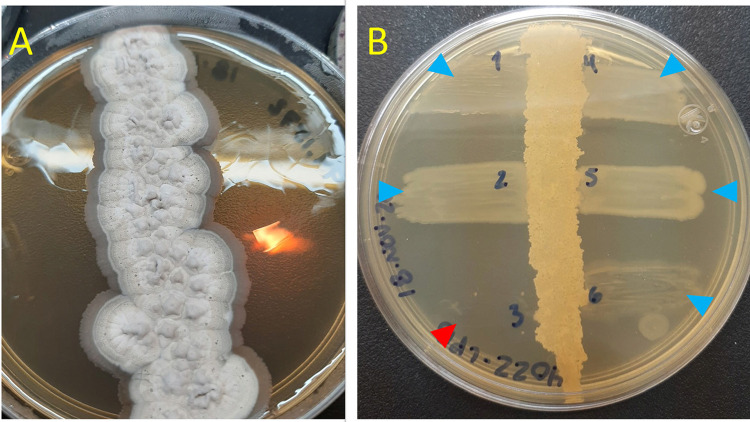
Examples of the results of the antimicrobial activity assay: A) *Streptomyces albus* 94.01570 inhibited the growth of all strains tested; B) *Nocardia brasiliensis* 4022-LPB, blue triangles show no inhibition of 1. *Escherichia fergusonii* Sec 23, 2. *Klebsiella variicola*, H776 4. *K. pneumoniae* subsp*. ozaenae* H759, and 5. *K. pneumoniae* subsp. *Pneumoniae* H1064; red arrow shows inhibition of 3. *K. oxytoca* H793.

Regarding the antagonistic capacity of actinobacteria, all 15 strains showed positive antimicrobial activity. Notably, 53% of the strains inhibited the growth of *K. oxytoca* H793, and 40% inhibited *E. fergusonii* Sec23. Four strains, representing 27% of the sample, inhibited *K. pneumoniae* subsp. *ozaenae* H759, four strains (27%) inhibited *K. pneumoniae* sub. *pneumoniae* H1064, and only 13% of the strains inhibited *K. variicola* H776. The strain *Streptomyces albus* 94,1572 presented the highest antimicrobial activity , inhibiting the growth of the five multidrug-resistant bacteria evaluated. Figures 1 and 2 summarize the results of the antimicrobial activity.

Twelve strains of the genus *Nocardia* were identified: *N. brasiliensis* (50%), *N. otitidiscaviarum* (25%), *N. beijiniensis* (17%), and *N. nova* (7%). The remaining three strains (20%) corresponded to *Streptomyces albus* (Figure 2). The ranges of the sequences obtained were above 1400 bp, with similarity percentages higher than 98%.

Among the bacteria identified as *Streptomyces albus*, strain M904 presented all four biosynthetic systems, while strain *S. albus* 94.1572 presented two biosynthetic systems (NRPS-I and PKS-I) but with antimicrobial activity against all the multidrug-resistant strains evaluated. As shown in Figure 2, the PKS-I biosynthetic system was more frequent in *Streptomyces* strains but not in *Nocardia* strains.

## DISCUSSION

Our study results reveal valuable information on the presence of biosynthetic systems in pathogenic actinobacterial strains of clinical origin and suggest significant potential in the search for solutions to address the critical problem of antimicrobial resistance, especially in the context of neonatal sepsis. Four biosynthetic systems were identified: NRPS-I, PKS-I, PKS-II, and NRPS-II. Notably, the NRPS-I system was present in all strains studied; this suggests that this system may play a key role in these actinobacteria, as has been previously reported for *Nocardia* spp.^28^

The PKS-I and PKS-II systems were also present in some strains. Specifically, the PKS-I system was found mostly in the genus *Nocardia* (*N. brasiliensis* 4216, *N. nova* 4437, and *S. albus* M-904), whereas the PKS-II system was identified mainly in the genus *Streptomyces* (*S. albus* 94.1572, *S. albus* 04.01570, and *S. albus* M-904). These systems are related to synthesizing secondary natural products, many of which have bioactive properties^28,29^; this suggests that these strains may produce bioactive compounds with possible pharmacological applications. The NRPS-II system was present in only one strain, *S. albus* M-904. The low presence of this system in the study strains raises interesting questions about its function and relevance in the biotechnological and pathogenic context.

Specifically, actinobacteria *N. brasiliensis* 4216 and *S. albus* 94.1572 were shown to have biosynthetic systems related to the synthesis of secondary natural products, which are often bioactive compounds with antimicrobial properties. This correlates with the *in vitro* antimicrobial activity, in which *S. albus* 94.1572 showed outstanding activity against the five multidrug-resistant strains evaluated. The ability of *N. brasiliensis* 4216 and *S. albus* 94.1572 to effectively combat these pathogens suggests the production of potentially novel and valuable antimycobacterial compounds, as found in other strains of the same genera^30-32^.

*N. brasiliensis* 4216 and *S. albus* 94.1572 also showed activity against the two extended-spectrum beta-lactamase (ESBL) producing pathogens tested, *E. fergusonii* Sec 23 and *K. pneumoniae* subsp. *pneumoniae* H1064. If we consider that, for first-line empirical treatment of neonatal sepsis, the use of extended-spectrum beta-lactams such as amoxicillin and benzylpenicillin, in combination with gentamicin or cefotaxime-ma/ceftriaxone as second line is recommended^6^, the presence of species (such as those reported) have a direct impact on the conservative treatment recommended by WHO^6^.

There are several reports of increasing antibiotic resistance of *K. pneumoniae* and the emerging species *E. fergusonii* ^15,33^ in the hospital setting and at the neonatal intensive care unit (NICU) level^34-36^. With the many reports about the importance of treatment with β-lactams and gentamicin in this population, it is clear that alternative elements that can help with the increasingly less useful treatment at the neonatal level are needed. Hence, it is necessary to find novel therapeutic alternatives. The antimicrobial activity test demonstrated the potential of actinobacteria to inhibit the growth of multidrug-resistant strains implicated in neonatal sepsis, which was the study aim.

The actinobacteria were identified within the genera *Nocardia* (12 strains) and *Streptomyces* (three strains). Both have been shown to have biosynthetic potential for novel bioactive compounds^37-39^. *Nocardia* are known as pathogens of animals and humans that cause infections known as actinomycetoma and nocardiosis, and they are also saprophytes degrading organic matter^38,39^. Strains isolated from clinical cases are frequently investigated for their pathogenicity mechanism and less frequently to study their biosynthetic potential, as an important source of new drugs.

The genus *Streptomyces* is the largest producer of natural compounds, so it has been exploited in the pharmaceutical industry; however, it is now known that some species encode more than 20 biosynthetic clusters, many of which are silent. A recent study has employed *Streptomyces* in genetic engineering to produce and improve natural products^39^.

Although three *Streptomyces* strains of the same species, *S. albus*, were identified and analyzed by 16S rRNA gene sequencing, they showed different bioactivity. Only strain 94.1572 showed sufficient activity to inhibit the five multidrug-resistant strains, while strains M-904 and 94.01572 inhibited two and three multidrug-resistant strains, respectively. This may show us that there is genetic variability within this same species with diverse secondary metabolite production capacity. We propose specific studies on the intraspecies genetic variability of *S. albus* to exploit its biotechnological potential more accurately.

Antibiotic development is increasingly complex, time-consuming, and expensive. It can take over a decade and high economic costs to develop a new antibiotic treatment with an overall success rate of only 10%. Despite this, the urgency for new treatments against multidrug-resistant bacteria is increasing^11^. For future research, we recommend conducting studies on the bioactive compounds produced by these strains and biochemical profiles that determine the Minimum Inhibitory Concentration.

Finally, regarding the choice of various multidrug-resistant *Klebsiella* species used in this study, it is important to note that neonatal sepsis is regularly associated with the presence of *Klebsiella pneumoniae*, which is identified by automated methods so that other species within the *Klebsiella* genus are often not accurately identified in the clinical setting. Among these species, *Klebsiella variicola* has been identified as part of the bacteria associated with neonatal sepsis, although its accurate identification can be challenging in clinical practice^5^. Our study addresses this issue by including the evaluation of species such as *K. variicola* and *K. oxytoca,* which, although often misidentified, may also play a significant role in neonatal sepsis^16^.

This study demonstrates that actinobacterial strains of clinical origin can be considered promising sources of antimicrobial agents in the fight against resistant bacterial infections. *N. brasiliensis* 4216 and *S. albus* 94.1572 have the potential to provide effective therapeutic alternatives in the fight against severe and resistant bacterial infections, which have increased in Latin American countries.

## CONCLUSION

Antibiotic resistance poses a significant threat to human health worldwide, with a particularly devastating impact on vulnerable populations such as neonates. Neonatal sepsis remains a major cause of mortality in Latin America and elsewhere. Therefore, it is imperative to continue researching and developing new antibacterial treatments to address this challenge. Our work highlights the potential of the studied bacteria, which have demonstrated biosynthetic capabilities to produce pharmacologically relevant compounds. These findings open new possibilities for developing therapeutic alternatives against antibiotic-resistant bacteria and offer real hope in the fight against infectious diseases.
